# Assessing self-criticism and self-reassurance: Examining psychometric properties and clinical usefulness of the Short-Form of the Forms of Self-Criticizing/Attacking & Self-Reassuring Scale (FSCRS-SF) in Spanish sample

**DOI:** 10.1371/journal.pone.0252089

**Published:** 2021-05-24

**Authors:** Jaime Navarrete, Rocío Herrero, Joaquim Soler, Elisabet Domínguez-Clavé, Rosa Baños, Ausiàs Cebolla

**Affiliations:** 1 Department of Personality, Evaluation, and Psychological Treatments, University of Valencia, Valencia, Spain; 2 Department of Psychiatry, Hospital de la Santa Creu i Sant Pau, Universitat Autònoma de Barcelona (UAB), Barcelona, Spain; 3 Department of Pharmacology, Therapeutics and Toxicology, Universitat Autònoma de Barcelona (UAB), Barcelona, Spain; 4 CIBER of Physiopathology of Obesity and Nutrition (CIBEROBN), Madrid, Spain; University of Twente, NETHERLANDS

## Abstract

The Forms of Self-Criticizing/Attacking and Self-Reassuring Scale (FSCRS) was designed to measure self-criticism (SC) through Inadequate Self (IS) and Hated Self (HS) factors, as well as self-reassurance (RS). However, its long and short forms have yet to be validated in the Spanish Population. The present study examines the psychometric properties of the short form (FSCRS-SF) and its clinical usefulness in a sample of 576 adult individuals, 77 with psychiatric disorders and 499 without. Non-clinical participants were split according to their previous experience with meditation (active meditators, *n* = 133; non-active meditators, *n* = 41; and non-meditators, *n* = 325) and differences between these subgroups were explored. Additionally, a subsample of 20 non-clinical participants took part in a mindfulness- and compassion- based intervention (MCBI) to assess the usefulness of the scale as an outcome measure. Results confirmed the original three-factorial structure, good internal consistency, acceptable test-retest reliability, and a pattern of correlations consistent with previous literature. Regarding differences between groups, the clinical subsample showed significant higher SC and lower RS levels than non-clinical participants and active meditators had significant lower IS and higher RS levels than non-meditators. Participants who participated in the MCBI showed significant RS improvement and a decrease in IS and HS levels. Moreover, a hierarchical multiple regression showed that RS made a significant predictive contribution to distress at three months’ time. In conclusion, results show that the Spanish version of the FSCRS-SF is a reliable and valid measure of SC and RS in non-clinical populations and an adequate instrument to detect changes after MCBIs.

## Introduction

Motivated by the desire to overcome the limitations of disorder-specific therapies, the transdiagnostic approach is gaining relevance in regard to treating mental health problems [1–4]. According to the transdiagnostic model, emotional disorders (i.e., depressive and anxiety disorders) have common aspects or factors underlying the symptomatology [5]. In this framework, self-criticism (SC) belongs to the family of intrusive or repetitive thoughts, and has been identified as a vulnerability factor for psychopathology and a transdiagnostic process of many mental health problems [6]. It underlies the development and maintenance of depressive [7–11], anxiety [12,13], and psychotic symptoms [14], eating disorders [15–18], non-suicidal self-injury [19], and suicidality [20,21]. Furthermore, SC also interacts with the evolution of the treatment, given that higher levels of baseline patients’ SC predict less symptom reduction [22].

The cognitive-evolutionary approach proposed by Gilbert [23] suggests that SC is a form of interaction between self-parts, which involves constantly expressing intense hostility and disrespect from a part of the self towards another. According to this model, SC can take the form of a sense of internal inadequacy because of mistakes (Inadequate Self), or an aggressive/destructive/pathological response to the self after failure (Hated Self). These forms of SC are the result of an apparent desire for self-improvement or the persecution of oneself as a form of personal revenge [24].

Moreover, Gilbert et al. [24] defined self-reassurance as an adaptive self-to-self interaction, specifically as the ability to bring kindness, caring, and peacefulness to the self when things go wrong. This self-reassurance ability is highly correlated to compassion [25], since both are adaptive forms of relating to the self in the context of difficult life struggles. In addition, self-reassurance seems to be an independent factor, instead of the opposite end of a SC dimension, according to several factor-analytic and fMRI studies [19,26,27]. Finally, it is a buffer against the development of psychopathology and fosters well-being [28–31].

Compassion is rooted in a care-giving mentality and is subject to change by mind-training procedures, which in turn reduces SC by developing a compassionate understanding of one’s situation [32]. Hence, SC has been used as an outcome for different interventions, especially those that include loving-kindness and compassion meditations [e.g., 33–37]. These meditations are part of the ‘relation orientation’ constructive family, which aim to strengthen healthy psychological patterns by nurturing prosocial qualities [38]. Similarly, cultivation of attentional regulation mechanisms (mindfulness) improves attention, body awareness (ability to focus on internal sensory experiences), reappraisal (positive reconstruction of stressful events), non-reactivity (non-reactive response to inner experience), and a detached perspective of the self [39,40]. Thus, attentional and constructive meditation practice leads to low levels of SC [23,41], though little is known about the evolution of SC over time, after decreasing or abandoning the meditation practice.

Based on the different models, researchers have developed several self-report questionnaires to assess SC, though to the best of our knowledge none of them have ever been validated to Spanish. Rose and Rimes [42] developed a systematic review to evaluate their measurement properties. These authors only recommended the use of the Forms of Self-Criticizing/Attacking and Self-Reassuring Scale [24] and the Self-Critical Rumination Scale [43] in future research taking into account their good psychometric properties and the high methodological quality of their validation studies.

### Forms of Self-Criticizing/Attacking and Self-Reassuring Scale (FSCRS)

Gilbert et al. [24] developed the 22-item FSCRS to focus on the concrete forms in which people engage in self-attacking behaviors when things go wrong for them, which previous instruments have failed to measure. They confirmed the Inadequate Self (IS) and the Hated Self (HS) factors and included a subscale for measuring the Reassured Self (RS). These three factors showed excellent reliability (IS Cronbach’s α = 0.90, HS Cronbach’s α = 0.86, and RS Cronbach’s α = 0.86).

In subsequent years, FSCRS has shown to be robust and reliable in both clinical and non-clinical populations in a range of different countries [44–48]. For instance, it has been used in the diagnosis of patients with borderline personality disorder (BPD) to examine the potential relationship between SC and recovery [49] and to evaluate the effects of a compassion-based intervention [33]. Furthermore, the FSCRS has been used to explore the phenomenology of SC in patients with eating disorders (ED) [e.g., 50,51] and SC proved to be a strong predictor of eating disorder symptoms [15].

Recently, a 14-item version (FSCRS-SF) was generated to minimize response burden and increase response rates in studies that require multiple assessments [52]. Sommers-Spijkerman et al. [52] tested its psychometric properties in a Dutch community sample and the three-factor model of the full FSCRS was replicated. The short form demonstrated acceptable internal consistency. Nevertheless, test-retest reliability was not acceptable for IS and HS scores, in contrast to the findings of Castilho et al. [44] with the full FSCRS. Moreover, the characteristics of the sample hinder generalization of the findings due to an underrepresentation of males and people with lower educational levels. Finally, the short form was not used as a stand-alone instrument as the extended version was. To this day, the study of Sommers-Spijkerman et al. [52] is the only one that has tested psychometric features of the short form.

### Spanish validation of the forms of Self-Criticizing/Attacking and Self-Reassuring Scale-Short Form

In the present moment, neither the full nor the short form of the FSCRS has been validated in the Spanish population. Given that the study of Sommers-Spijkerman et al. [52] shows similar psychometric properties between both forms, the current study aims to translate and validate the 14-items FSCRS into Spanish in a large sample. The main reason to validate the shortened version of the FSCRS is to facilitate the assessment of SC and RS in research contexts, in which multiple measures are commonly used. The validity of the FSCRS-SF for discriminating between non-clinical and clinical samples. Moreover, we aimed to assess the additional utility of using FSCRS-SF for predicting a global mental health index, studying its potential role as a mediator between meditation practice and distress, and its usefulness as an outcome measure after a mindfulness- and compassion- based intervention (MCBI).

We collected data from three samples of participants, including a non-clinical sample composed of frequent meditators, non-active meditators, and non-meditators, a clinical sample of patients with BPD and patients with ED, and a social worker sample. Firstly, factor structure was evaluated in the non-clinical sample; we hypothesized that the three-factor model would show adequate fit as well as it did in the Dutch population. Secondly, we expected similar evidence of its reliability and construct validity in the non-clinical sample compared to both the full and short original versions. That is, SC factors were expected to be positively associated with self-critical thinking, depression, anxiety, and somatization symptoms and negatively associated with self-compassion. Conversely, RS was expected to show a negative association with self-critical thinking and psychopathology symptoms, and a positive association with self-compassion. Reliability was also studied in the clinical sample. The third hypothesis stated that non-clinical participants who meditate will show significantly lower levels of IS and higher RS than both those who have meditation experience but did not practice during the last year and non-meditators. Regarding this hypothesis, we did not expect differences in HS scores among these non-clinical subgroups because the HS subscale may not distinguish between non-clinical test-takers [45]. However, we expected that the clinical sample of BPD and ED patients would show significantly higher SC, especially HS levels, and lower RS than the non-clinical sample. We expected that the FSCRS-SF factors would significantly predict distress at three months’ time in the non-clinical sample, would mediate the effects of meditation practice on non-clinical participants’ general distress, and finally that the scale would be able to detect significant improvements in SC and RS after an MCBI in the social worker sample.

## Materials and methods

### Participants

The Ethics Committee of Research in Humans of the Ethics Commission in Experimental Research of the University of Valencia approved the procedure (H1539699805131). We obtained written consent from all participants involved in this study. The non-clinical sample was composed of 499 adults living in Spain (122 men and 377 women), ranging from 18 to 75 years of age (*M* = 31.08, *SD* = 12.55). From them, 22 males and 97 females, ranging from 18 to 67 years of age (*M* = 31.09; *SD* = 12.11), composed the subsample for the analysis of test-retest reliability. Adult patients diagnosed with BPD (*n* **=** *36*) and ED (*n =* 41) were recruited at the Hospital de la Santa Creu i Sant Pau (Barcelona, Spain) and made up the clinical sample (4 men and 73 women), ranging from 17 to 65 years of age (*M* = 31.03, *SD* = 11.03). Finally, a sample of 20 social workers (all women) took part in a MCBI, their age ranged from 25 to 56 years of age (*M* = 39.65, *SD* = 8.29). Participant characteristics are listed in Table 1.

**Table 1:** Sociodemographic characteristics of participants.

	Non-clinical sample		Clinical sample	
Variable	*n*	%	*n*	%
**Gender**				
Female	377	75.55	73	94.8
Male	122	24.45	4	5.2
**Marital status**				
Single	364	72.95	NA	NA
Married/partnered	99	19.84	NA	NA
Divorced/widowed	36	7.21	NA	NA
**Highest educational level**				
Middle school	26	5.21	12	15.58
High school/some college	51	10.22	37	48.05
University or post-graduate degree	415	83.17	28	36.36
Other	7	1.40	0	0

NA = Not available.

Regarding the non-clinical sample, 174 reported some kind of previous meditation experience whereas 325 reported no experience at all. More specifically, 133 were ‘active meditators’ (i.e., they meditate at least 2 or 3 times per month) and 41 ‘non-active meditators’ who had not practiced meditation during the last year.

### Procedure

Participants were recruited among undergraduate students at the University of Valencia and Castellón (Spain), and throughout the autonomous community in general. The sample was recruited through advertisements (online ads in social media sites and internet forums) and flyers announcing the study to university students after their classes. The assessment was conducted between September (2019) and March (2020). Those who were interested in participating received an e-mail with a link to the online survey that was programmed on the LimeSurvey of the University of Valencia or a pen-and-paper questionnaire package. In total, 437 individuals completely filled out the battery and 62 filled it out partially (only the FSCRS-SF). Moreover, all of them received a second link to answer the FSCRS-SF (and psychopathology symptoms with BSI-18) 3 months after the assessment (119 participants). Additionally, BPD and ED outpatients from the Hospital de la Santa Creu i Sant Pau (Barcelona, Spain) were invited to voluntarily participate in the present study. Those who agreed answered the pen-and-paper survey *in situ*. Finally, a sample of 20 social workers participated in a blended-internet MCBI intervention and the FSCRS-SF was administered before and after this intervention. These participants were recruited from the College of Social Workers of Valencia. The MCBI was an 8-week program called Well-being training based on contemplative practices (WTCP; [53]). Based on contemplative positive psychology, understood as an “area of positive psychology that includes a range of techniques and conceptualizations developed by the contemplative sciences for the promotion of well-being through evidence-based strategies” [53] this training was developed to train skills that predict well-being supported by contemplative practices. The premise of the WTCP program is twofold: a) it is possible to train the skills and abilities to achieve a balanced and virtuous mind through the mental training involved in the practice of meditation and b) this process, in turn, will increase the probability of increasing one’s levels of psychological well-being. Each of these skills, based on Richard Davidson and Schuyler’s work [54], is developed throughout the WTCP program, generating different practices, tasks, and meditations.

### Measures

#### Forms of Self-Criticizing/Attacking and Self-Reassuring Scale—Short Form (FSCRS-SF)

Sommers-Spijkerman et al. [52] proposed a short version of the Forms of Self-Criticizing/Attacking and Self-Reassuring Scale (FSCRS). Permission from the original authors of the FSCRS-SF and The Compassionate Mind Foundation was obtained to reproduce and validate the FSCRS-SF into Spanish. Three independent English/Spanish speakers translated this 14-item short form to Spanish, and subsequently an independent native English speaker translated it back to English. After discussing the discrepancies, the Spanish-language version was adapted until it was equivalent to the original FSCRS-SF. It comprises three independent scales (IS, HS, and RS) with five, four, and five items, respectively. Participants respond to statements, which ask about how they react ‘when things go wrong’, on a 5-point Likert scale ranging from 0 (Not at all like me) to 4 (Extremely like me). Higher scores indicate a greater sense of inadequacy (score 0–20), self-hate (score 0–16), and self-reassurance (score 0–20). See final Spanish version of the questionnaire in the online supporting information section (S1 File).

#### Self-Critical Rumination Scale (SCRS)

The SCRS comprises 10 items and a 4-point Likert scale ranging from 1 (not at all) to 4 (very well) to assess SC [43]. We used an ad hoc Spanish-language version of the SCRS, which is in process of validation in a parallel study. The scale has a single-factor structure and showed excellent internal consistency [43]. Higher scores indicate a greater negative thinking that devaluates the self (score 10–40). The SCRS demonstrated excellent internal consistency (Cronbach’s α = 0.91; McDonald’s ω = 0.91) in the present sample.

#### Self-Compassion Scale Short-Form (SCS-SF)

The SCS-SF is a 12-item measure assessing self-compassion [55]. We used the Spanish validated version, which reported good internal consistency [56]. Items are scored on a 5-point Likert scale from 1 (Almost never) to 5 (Almost always), and all scores are added to form a total score. We also calculated separate scores for the Positive (Self-Kindness, Common Humanity, and Mindfulness) and Negative (Self-Judgement, Isolation, and Over-identified) subscales following recent recommendations about the interpretation and scoring of the scale [57,58]. The SCS-SF demonstrated good internal consistency (Total score Cronbach’s α = 0.86, McDonald’s ω = 0.86; Positive Subscale Cronbach’s α = 0.78, McDonald’s ω = 0.79; Negative Subscale Cronbach’s α = 0.83, McDonald’s ω = 0.83) in the present sample.

#### The Brief Symptom Inventory-18 (BSI-18)

Depression, anxiety, and somatization symptoms were assessed using the 18-item version of the BSI [59]. Participants rate the frequency of depressive symptoms (BSI-D, 6 items, score 0–24), anxiety symptoms (BSI-A, 6 items, score 0–24), and somatization (BSI-S, 6 items, score 0–24) over the past week on a 5-point Likert scale from 0 (Not at all) to 4 (Always). Moreover, the questionnaire offers a global scale of general distress (BSI-T) ranging from 0 to 108. The BSI-18 has good dimensional structure and reliability in the Spanish population [60]. The internal consistency of the subscales (BSI-D Cronbach’s α = 0.88, McDonald’s ω = 0.89; BSI-A Cronbach’s α = 0.81, McDonald’s ω = 0.82; BSI-S Cronbach’s α = 0.83, McDonald’s ω = 0.83) and the global index (BSI-T Cronbach’s α = 0.93, McDonald’s ω = 0.93) have been found to be good.

### Data analysis

Descriptive statistics, Confirmatory Factor Analysis (CFA), internal consistency, intercorrelations between FSCRS-SF subscale scores, test-retest reliability, convergent and divergent validity, a one-way between-groups multivariate analysis (MANOVA), a paired-samples t-test, a hierarchical multiple regression, and mediation analyses were all calculated starting with the descriptive statistics of the items in the non-clinical and clinical samples (see Table 2). CFA and internal consistency were computed using JASP (Version 0.12.1) while test-retest, validity analysis, MANOVA, multiple regression, and mediation analysis were conducted in IBM SPSS Statistics for Windows (Version 26).

**Table 2:** Descriptive statistics of the FSCRS-SF items.

	Non-clinical sample (*n* = 499)						Clinical sample (*n* = 77)					
Items	*M*	*Mdn*	*Md*	*SD*	Skew	Kurt	*M*	*Mdn*	*Md*	*SD*	Skew	Kurt
1	2.16	2	3	1.01	-0.20	-0.65	1.79	2	1	1.06	0.36	-0.55
2	1.27	1	1	1.11	0.57	-0.54	3.04	3	4	1.08	-1.10	0.71
3	2.05	2	3	1.12	-0.24	-0.81	1.45	1	1	1.12	0.52	-0.40
4	1.58	1	1	1.23	0.31	-1.01	3.13	4	4	1.06	-1.02	0.36
5	2.95	3	4	1.07	-0.90	0.06	1.32	1	0	1.14	0.42	-0.63
6	0.62	0	0	1.13	1.78	1.95	1.81	1	0	1.57	0.23	-1.52
7	0.46	0	0	0.90	2.02	3.33	2.23	2	4	1.44	-0.21	-1.35
8	3.10	3	4	1.07	-1.21	0.83	1.81	2	3	1.29	0.07	-1.19
9	0.91	1	0	1.11	1.15	0.48	2.16	2	2	1.38	-0.23	-1.07
10	1.09	1	0	1.13	0.92	0.06	2.58	3	3	1.20	-0.51	-0.57
11	0.70	0	0	1.01	1.51	1.60	2.21	2	4	1.54	-0.18	-1.46
12	1.19	1	1	1.07	0.69	-0.26	2.88	3	4	1.20	-0.70	-0.59
13	1.60	1	1	1.31	0.42	-1.01	3.47	4	4	0.82	-1.53	1.57
14	2.71	3	3	1.09	-0.55	-0.55	2.04	2	2	1.28	0.04	-1.02

FSCRS-SF = Forms of Self-Criticizing/Attacking and Self-Reassuring Scale-Short Form. M = mean; Mdn = median; Md = mode; Skew = Skewness; Kurt = Kurtosis. Items scores ranging from 0 (not at all like me) to 4 (extremely like me).

#### Factor structure

Factor structure was assessed with the data collected from the non-clinical sample. Regarding CFA, preliminary analysis was conducted to ensure no violation of the assumptions of normality, linearity, multicollinearity, and absence of outliers and residuals. Distribution of some item scores were significantly skewed or highly kurtotic, therefore the robust maximum likelihood method was employed to estimate the three-factor model that had been hypothesized. Several fit criteria were computed such as the χ^2^ statistic, the Standardized Root Mean Square Residual (SRMR), the Root Mean Square Error of Approximation (RMSEA), Comparative Fit Index (CFI), and Tucker-Lewis Fit Index (TLI) to determine the good fit between the model and the data. Following Hu and Bentler’s [61] recommendations regarding cut-off criteria, we used the combination of the SRMR ≤ 0.08, RMSEA ≤ 0.06, and CFI and TLI close to 0.95 to determine good fit between the model and the data. Standardized factor loadings ≥.50 were considered acceptable and ≥.70 were considered strong [62].

#### Internal consistency and intercorrelations between FSCRS-SF subscale scores

To assess subscale reliability, internal consistency was assessed using Cronbach’s α [63] and McDonald’s ω [64]. Additionally, we calculated intercorrelations between the FSCRS-SF subscale scores using Pearson’s correlation coefficient. Both of them were assessed with the data collected from the non-clinical and clinical samples.

#### Test-retest reliability and convergent and divergent validity

Test-retest reliability and convergent and divergent validity were assessed with the data collected from the non-clinical sample. Pearson’s correlation was used by correlating the FSCRS-SF scores with the SCRS, SCS-SF, BSI-18, and post-FSCRS-SF ones. Effect size guideline for interpreting small, medium, and large correlations were 0.15, 0.25, and 0.35, respectively [65].

#### Known-groups validity

A MANOVA was performed to investigate clinical differences in SC and RS. FSCRS-SF factors were the dependent variables. Data from the non-clinical and clinical samples were used. The independent variable had four levels: active meditators, non-active meditators, participants without meditation experience, and clinical participants. We conducted post hoc analyses using Tukey Honestly Significant Difference (HSD) test to identify where the potential significant differences lie. Preliminary assumption testing was conducted to check for normality, linearity, univariate and multivariate outliers, homogeneity of variance-covariance matrices, and multicollinearity, with no serious violations noted.

#### Sensitivity to change

A paired-samples t-test was conducted to evaluate the impact of the MCBI intervention on social workers’ scores. Eta squared was the effect size statistical measure calculated for both MANOVA and t-test. It was interpreted following Cohen’s [66] guidelines: .01 (small effect), .06 (moderate effect), and .14 (large effect).

#### Predictive validity

Hierarchical multiple regression was used to assess the ability of the FSCRS-SF measure to predict levels of non-clinical participants’ general distress (BSI-T), after controlling for the influence of baseline distress. Preliminary analyses were conducted to ensure no violation of the assumptions of normality, linearity, multicollinearity, and homoscedasticity.

#### Mediation analysis

Multiple mediation analyses, or simultaneous mediation by multiple mediators, were carried out with the data collected from the non-clinical sample following the methodology described by Hayes [67] from the PROCESS macro (version 3.4.1), choosing model 4. Thus, we studied the FSCRS-SF factors role as mediators between meditation practice and BSI-T scores at baseline. The confidence interval (CI) for the indirect effect was a percentile bootstrap 95% interval based on 5000 samples. CI that did not contain the zero-value indicated a significant indirect effect.

## Results

### Factor structure

**Fig 1:**
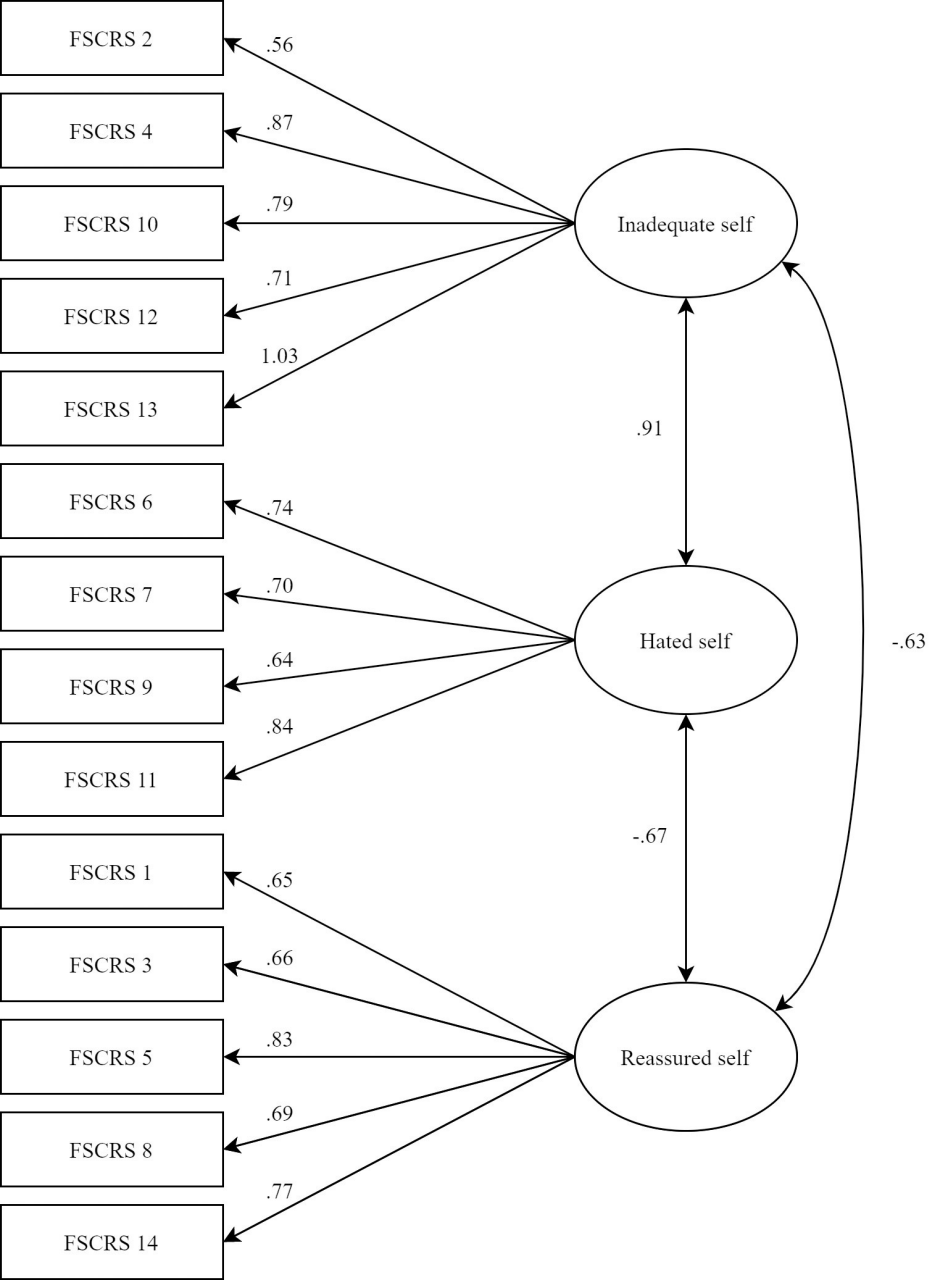
Confirmatory factor analysis model of the Forms of Self-Criticizing/Attacking and Self-Reassuring Scale-Short Form.

The hypothesized model with coefficients presented in standardized form is shown in Fig 1. It was tested and support was found for the three-factor model, χ^2^ (74, *N* = 499) = 238.13, *p* < .001, SRMR = 0.04, RMSEA = 0.06, CFI = 0.94, and TLI = 0.93. Factor loadings were acceptable, ranging from .56 to 1.03.

### Internal consistency and intercorrelations between FSCRS-SF subscale scores

Regarding reliability, the internal consistency of the FSCRS-SF subscales was adequate for both clinical and non-clinical samples (see Table 3). The intercorrelation of the three FSCRS-SF subscales were large for both non-clinical (*|r|* ≥ .52) and clinical samples (*|r|* ≥ .63). Table 3 shows Cronbach’s α, McDonald’s ω, and intercorrelations of the FSCRS-SF subscales.

**Table 3:** Pearson intercorrelations and internal consistency of the FSCRS-SF subscales in clinical and non-clinical samples.

	Non-clinical sample (*n* = 499)			Clinical sample (*n* = 77)		
FSCRS-SF subscales	Inadequate self	Hated self	Reassured self	Inadequate self	Hated self	Reassured self
Inadequate	-	-	-	-	-	-
self	.73	-	-	.69	-	-
Hated self	-.52	-.52	-	-.63	-.67	-
Reassured self						
Cronbach’s α	.80	.80	.81	.79	.82	.80
McDonald’s ω	.81	.81	.81	.80	.82	.82

FSCRS-SF = Forms of Self-Criticizing/Attacking and Self-Reassuring Scale-Short Form. All correlations were significant at *p* < .01.

### Test-retest reliability and convergent and divergent validity

Test-retest reliability at 3 months was *r* = .78 for IS subscale, *r* = .73 for HS subscale, and *r* = .66 for RS subscale (all with *p* < .01), which supports temporal stability of FSCRS-SF scores. Table 4 shows correlations between FSCRS-SF with SCRS, SCS-SF, and BSI-18. All correlations were large, significant, and in the expected direction, supporting the convergent and divergent validity of the FSCRS-SF.

**Table 4:** Convergent and divergent validity of the FSCRS-SF scales.

			FSCRS-SF		
Measure	*M*	SD	Inadequate self	Hated self	Reassured self
**SCRS**					
Total score	20.94	7.23	.74**	.60**	-.43**
**SCS-SF**					
Positive subscale	18.26	5.01	-.49**	-.45**	.61**
Negative subscale	15.39	5.50	.78**	.60**	-.46**
Total score	38.88	9.14	-.74**	-.61**	.61**
**BSI-18**					
Depressive symptoms	5.55	5.48	.60**	.59**	-.48**
Anxiety symptoms	5.01	4.62	.47**	.38**	-.35**
Somatization	3.86	4.62	.36**	.35**	-.28**
General distress	14.43	13.10	.55**	.51**	-.43**

*N* = 499. FSCRS-SF = Forms of Self-Criticizing/Attacking and Self-Reassuring Scale-Short Form; SCRS = Self-Critical Rumination Scale; SCS-SF = Self-Compassion Scale Short-Form; BSI-18 = The Brief Symptom Inventory-18.

**p < .01.

### Known-groups validity

With the use of Wilks’ criterion, the combined FSCRS-SF factors were significantly affected by group condition, *F* (9, 1370.35) = 27.24, *p* < .001; Wilks’ Lambda = 0.67; *η*^*2*^ = 0.13. When the results for the dependent variables were considered separately, there were statistically significant differences in IS, HS, and RS (see Table 4). Post hoc comparisons using the Tukey HSD test indicated that the clinical sample had statistically significant higher IS (*p* < .001) and HS (*p* < .001) and lower RS (*p* < .001) mean scores than all non-clinical subgroups. Moreover, there were statistically significant differences in IS (*p* = .013) and marginally significant differences in RS (*p* = .050) between the mean scores of active meditators and participants without meditation experience. Table 5 shows means, standard deviations, and one-way analyses of variance in FSCRS-SF factors.

**Table 5:** Means, standard deviations, and one-way analyses of variance in FSCRS-SF subscales.

Measure	Active meditators (*n* = 133)	Non-active meditators (*n* = 41)	Non-meditators (*n* = 325)	Clinical sample (*n* = 77)	*F* (3, 565)	*η*^*2*^
**FSCRS-SF**						
Inadequate self	6.08 (4.07)	5.61 (3.77)	7.15 (4.52)	15.17 (3.99)	87.49***	.32
Hated self	2.47 (3.30)	2.02 (2.82)	2.88 (3.30)	8.47 (4.77)	60.68***	.24
Reassured self	13.71 (4.01)	13.83 (3.47)	12.59 (4.03)	8.39 (4.42)	31.49***	.14

All values represent mean scores (standard deviations in parenthesis); FSCRS-SF = Forms of Self-Criticizing/Attacking and Self-Reassuring Scale-Short Form. For non-clinical sample (i.e., active, non-active, and non-meditators): IS (*M* = 6.73; *SD* = 4.37); HS (*M* = 2.70; *SD* = 3.27); RS (*M* = 12.97; *SD* = 4.01).

*** *p* < .001.

### Sensitivity to change

Regarding the subsample (*n* = 20) that underwent the MCBI, there was a statistically significant decrease in IS scores from Time 1 (*M* = 7.40, *SD* = 3.57) to Time 2 (*M* = 5.80, *SD* = 2.48), *t* (19) = 2.20, *p* = .04 (two-tailed) with eta squared (.20) indicating a large effect size. In terms of HS, there was no statistically significant decrease in scores from Time 1 (*M* = 1.75, *SD* = 1.48) to Time 2 (*M* = 1.25, *SD* = 1.16), *t* (19) = 1.75, *p* = .096 (two-tailed) and eta squared (.14) indicated a large effect size. Finally, there was a statistically significant increase in RS scores from Time 1 (*M* = 13.50, *SD* = 3.28) to Time 2 (*M* = 15.80, *SD* = 2.91), *t* (19) = -3.19, *p* = .005 (two-tailed) with eta squared (.35) indicating a large effect size.

### Predictive validity

Table 6 shows the regression coefficients for the hierarchical multiple regression. BSI-T baseline scores were entered during Step 1, explaining 49.5% of the variance in general distress scores at three months’ time. After entering the IS, HS, and RS factors, the total variance explained by the model as a whole was 52% *F* (4, 109) = 29.55, *p* < .001. The SC measures explained an additional 2.6%, after controlling for baseline scores, *R* squared change = .026, *F* change (3, 109) = 1.95, *p* = .126. In the final model, RS was the only FSCRS-SF factor to reach statistical significance with a beta = –.17 (*p* = .041).

**Table 6:** Hierarchical regression results for global index of general distress at three months’ time.

	Model				
Scales	*B*	*SE*	β	*t*	*p*
**Step 1**					
Constant	4.65	1.35		3.44	.001
BSI-T (baseline)	0.73	0.07	0.70	10.47	.000
**Step 2**					
Constant	12.47	4.72		2.64	.009
BSI-T (baseline)	0.73	0.08	0.62	7.61	.000
FSCRS-SF (Inadequate self)	0.19	0.32	0.06	0.59	.560
FSCRS-SF (Hated self)	-0.19	0.42	-0.05	-0.46	.650
FSCRS-SF (Reassured self)	-0.58	0.27	-0.17	-2.07	.041

BSI-T = Brief Symptom Inventory Total Score; FSCRS-SF = Forms of Self-Criticizing/Attacking and Self-Reassuring Scale-Short Form.

### Mediation analysis

**Fig 2:**
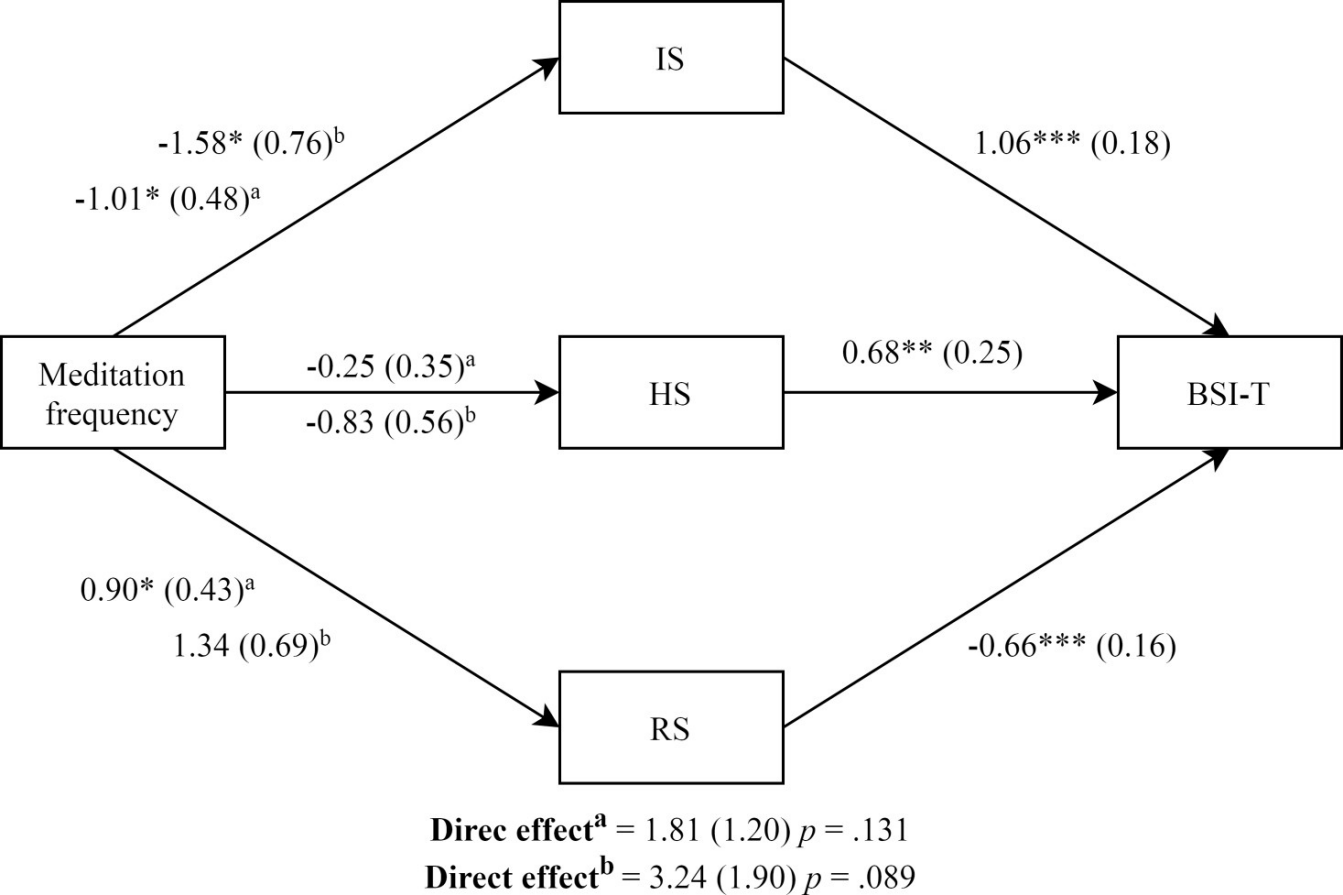
Influence of meditation practice on general distress through self-criticism. IS = Inadequate self; HS = Hated self; RS = Reassured self. a = Meditators vs. non-meditators. b = Non-active Meditators vs. non-meditators * p < .05. ** p < .01. ***p < .001.

As can be seen in Fig 2 and Table 7, participants who actively meditate, even those who did not practice during the last year, reported a lower distress level than participants without meditation experience, mediated by IS and RS levels. There was no evidence that active/non-active meditators and those without meditation experience influenced baseline BSI-T scores independent of its effect on IS and RS.

**Table 7:** Mediation analysis: Indirect effects of meditation on distress through self-criticism and self-reassuring.

				95% CI	
Antecedent	Mediator	Coeff.	*SE*	*LL*	*UL*
Active meditators	**IS**	-1.07	0.54	-2.23	-0.09
vs	**HS**	-0.17	0.27	-0.76	0.33
Non-meditators	**RS**	-0.59	0.33	-1.33	-0.02
Non-active meditators	**IS**	-1.67	0.72	-3.19	-0.34
vs	**HS**	-0.57	0.37	-1.39	0.03
Non-meditators	**RS**	-0.88	0.47	-1.94	-0.09

IS = Inadequate self; HS = Hated self; RS = Reassured self; SE = standard error; CI = confidence interval; *LL* = lower limit; *UL* = upper limit.

## Discussion

To our knowledge, the current study is the first validation study of the FSCRS-SF for the Spanish population and the second that validates it after the original work of Sommers-Spijkerman et al. [52]. Our main contribution was to test the FSCRS-SF as a stand-alone instrument for the first time, i.e., to directly administer the FSCRS as short form. Furthermore, it was assessed in a clinical sample composed of BPD and ED patients, for whom measurement and treatment of SC is essential. The aim was to examine the psychometric properties of the Spanish validation of the short form of the FSCRS, to explore differences in SC and RS among the general population with different levels of meditation experience and clinical patients, to assess its usefulness as an outcome measure after an MCBI, and to explore the mediator and predictive role of SC and RS on psychopathology symptoms. Its factorial structure, internal consistency, convergent, divergent, known-group, and predictive validity were examined.

The three-dimensional factor structure provided a very good fit in the non-clinical sample. This result supports the previous findings of Sommers-Spijkerman et al. [52] regarding the three-factor solution and rationale of the cognitive-evolutionary model [68]. In addition, the three-dimensional model provides a better fit than the unidimensional and bifactorial ones according to the results of the confirmatory factor analyses of the FSCRS in clinical and non-clinical samples [44,45,48,69–71]. This is in contrast with a large intercultural study which has shown that a two-factor model of RS and SC (combining IS and HS) had a superior fit, although the three-factor one was acceptable too [46]. Then, following the parsimony principle in factor analysis, it seems that professionals should choose the simpler model. However, Gilbert’s [23] theoretical understanding of SC has important implications for clinical practice, since it offers a more accurate description of SC manifestation in non-clinical and clinical samples. So, to the extent that both factor structures are acceptable, researchers and clinicians should choose those most relevant to their context.

Regarding reliability, internal consistency analyses showed the FSCRS-SF as a reliable self-report measure. Reliability coefficients are higher than those obtained in the Dutch version ([52]; α = .52-.72; ω = .49-.72) and these results are in line with previous studies of the long form [44,46]. Furthermore, the test-retest reliability was acceptable for IS and HS scores and questionable for RS scores, which means that these subscales are reasonably stable considering the three-month interval. It allows the assessment of the effectiveness of interventions aimed at reducing SC. As proof of this, participants who participated in the MCBI showed significant improvements in RS and a decrease in IS and HS levels. Along this line, the FSCRS has been able to detect SC and RS changes in a variety of interventions, such as compassion-based interventions [e.g., 33,72] and mindfulness-based interventions [e.g., 34].

Our results indicate a clear relationship between SC facets and negative psychological health. These correlation patterns are consistent with previous findings regarding IS and HS factors, which have been associated with high levels of depression, anxiety, stress, pessimism, isolation, shame, eating-related difficulties, body mass index, and facial-emotion recognition difficulties [44,48,72–75]. Along these lines, RS has been associated with high levels of self-compassion [76], dispositional optimism [44], weight-related positive affect, and overall mental well-being [77]. Furthermore, BPD and ED patients showed significant higher SC and lower RS levels than non-clinical participants, thus indicating that the FSCRS-SF is able to discriminate between clinical groups that are known to differ in these two variables. A range of disorders, which include BPD and ED, are associated with high SC and low RS [69], though further studies are needed to describe mechanisms through which SC works in these pathologies [6]. In the case of BPD, SC and self-invalidation [78] are terms that, without having the exact same meaning, have huge overlap. Moreover, the roles of invalidation and self-invalidation in the development of BPD have been already studied [79,80]. In this regard, Perkins et al. [81] have recently described how SC predicts nonsuicidal self-injury behaviors at two months’ time in an ED sample. In addition, high SC has been associated with affective variability in BPD patients [82].

As mentioned previously, SC is sensitive to meditation training (e.g., WTCP [53]), in particular to meditation-based interventions that foster compassion, which is the theoretically proposed change mechanism underlying the decreasing of SC [e.g., 23,37,83]. In this regard, a recent meta-analysis has shown that self-compassion interventions are effective for decreasing self-criticism [84]. For instance, Compassion-Focused Therapy (CFT) focus on cultivating feelings of safeness and reassurance toward oneself in order to reduce SC [23]. As well, the Mindful Self-Compassion (MSC; [83]) and Loving-Kindness Meditation (LKM; [37]) programs teach people to be kinder and more compassionate toward themselves through meditation. However, not only interventions that explicitly teach compassion (e.g., CFT, MSC, and LKM) are effective to enhance it, but also mindfulness-based interventions, which teach compassion implicitly (e.g., Mindfulness-based Stress Reduction program) are effective too [85,86]. Although the implicit teaching of self-compassion might have a lower impact on it [87]. In fact, meditation experience (time of regular practice) was associated with lower levels of repetitive negative thinking, a similar psychological process to SC, being this association mediated by both self-compassion and mindfulness [88]. However, differences in SC between active meditators and meditators who abandoned the practice have not been previously investigated. We hypothesized that participants who usually meditate will show significant lower levels of IS and higher RS than both those who have meditation experience but did not practice during the last year and non-meditators. Our findings show that individuals who usually meditate and non-active meditators had no significantly different IS and RS levels. However, active meditators had significantly lower IS and higher RS levels than non-meditators. Interestingly, mediation analysis showed that both subsamples had less internal inadequacy by mistakes and more self-reassuring responses than participants without meditation experience, which in turn led them to feel less distress than non-meditators. Altogether, these results suggest that some mechanisms that could be learnt and developed through meditation [39] are temporally stable and may protect from SC, even for those who stopped meditating a year ago. Further studies should be designed to analyze this interesting result. In this line, Sommers-Spijkerman et al. [31] examined the potential mediator role of RS and IS in explaining the underlying mechanisms through Compassion-Focused Therapy (CFT). Their results showed that participants who received CFT intervention (compared to wait-list participants) had more RS and less IS after the intervention, in turn leading to more well-being.

Our findings also show that RS was the only FSCRS-SF factor that made a significant predictive contribution to distress at three months’ time. This result suggests that the ability to self-reassurance when things go wrong is better predicting psychopathology than SC. This supports the idea that RS is an independent factor for self-defense against SC [e.g., 89], meaning that its presence (or lack) determines the effect of SC. In this regard, RS was found to be a buffer of IS on depressive symptomatology [30]. That is, IS was weakly associated to depressive symptoms in individuals with high RS while this relationship was stronger and significant for low-RS ones.

### Limitations and future research

Regarding the present study, some limitations deserve to be addressed. First of all, the combination of online and in-person recruitment might have biased the results. In addition, the design of the present study is mainly cross-sectional, so the causality suggested by the mediation analysis should be complemented with future longitudinal studies. Third, participants were classified in the levels of the independent variable ‘meditation experience’ according to brief reported information of their previous practice. Finally, sample size could be larger and subsamples more balanced to increase the power of the analysis and to conduct measurement invariance evaluation, although ours was above the minimum for the implemented psychometric techniques. Also connected to this issue, it is worth noting that the R squared change in the hierarchical multiple regression was small, although the predictive model including baseline distress and FSCRS-SF subscales was statistically significant.

Likewise, future research could test measurement invariance of FSCRS-SF subscales across non-clinical and clinical samples. In addition, other Spanish-speaking countries should evaluate our Spanish language version taking into account expressions that may have a different meaning. Finally, interventions with control condition to treat SC should confirm the usefulness of this short form to measure pre-post changes.

## Conclusions

In summary, the Spanish version of the FSCRS-SF is a valid and reliable instrument to measure IS, HS, and RS in non-clinical samples. The findings also show that the FSCRS-SF subscales distinguish between clinical and non-clinical individuals and predict distress at three months’ time. Moreover, IS and RS mediate distress among individuals with different frequency of meditation practice and this short form is an adequate outcome instrument to detect changes after an MCBI.

An implication for practice is thus offering the possibility to assess SC and RS with a shortened version of the FSCRS with all psychometric guarantees, which is especially useful in the research context. Moreover, our results add information about the maintenance of the effects of meditation practice on people’s ability to reassure themselves and be less self-critical. This gives hints about the planning of follow-up sessions in the context of compassion-based interventions, for instance.

## Supporting information

S1 File Spanish translation of the Forms of Self-Criticizing/Attacking and Self-Reassuring Scale-Short Form. (DOCX)
