# Supplementary material for: Assessing self-criticism and self-reassurance: Examining psychometric properties and clinical usefulness of the Short-Form of the Forms of Self-Criticizing/Attacking & Self-Reassuring Scale (FSCRS-SF) in Spanish sample
Source: PLoS One. 2021 May 24;16(5):e0252089. doi: 10.1371/journal.pone.0252089 (PMC8143420; doi:10.1371/journal.pone.0252089)
Supplement: S1 File — (DOCX) [file pone.0252089.s001.docx]

**Spanish translation of the Forms of Self-Criticizing/Attacking and Self-Reassuring Scale-Short Form.**

A continuación, hay una serie de pensamientos y sentimientos que la gente a veces tiene. Lee cada afirmación cuidadosamente y rodea el número que mejor describa cómo de cierta es cada afirmación para ti. Por favor utiliza la siguiente escala:

| En absoluto como yo | Un poco como yo | Moderadamente como yo | Bastante como yo | Extremadamente como yo |
| --- | --- | --- | --- | --- |
| 0 | 1 | 2 | 3 | 4 |

|  | **Cuando las cosas me van mal:** |  |  |  |  |  |
| --- | --- | --- | --- | --- | --- | --- |
| 1 | Soy capaz de recordar cosas positivas sobre mí mismo/a. | 0 | 1 | 2 | 3 | 4 |
| 2 | Me resulta difícil controlar la ira y la frustración hacia mí mismo/a. | 0 | 1 | 2 | 3 | 4 |
| 3 | Me resulta fácil perdonarme. | 0 | 1 | 2 | 3 | 4 |
| 4 | Una parte de mí siente que no soy lo suficientemente bueno/a. | 0 | 1 | 2 | 3 | 4 |
| 5 | A pesar de todo me gusta ser yo. | 0 | 1 | 2 | 3 | 4 |
| 6 | He llegado a estar tan enfadado/a conmigo mismo/a que he querido dañarme o herirme. | 0 | 1 | 2 | 3 | 4 |
| 7 | Tengo una sensación de repulsión hacia mí mismo/a. | 0 | 1 | 2 | 3 | 4 |
| 8 | A pesar de todo siento que soy digno/a de ser amado/a y aceptado/a. | 0 | 1 | 2 | 3 | 4 |
| 9 | Dejo de cuidarme. | 0 | 1 | 2 | 3 | 4 |
| 10 | Me regodeo en mis errores. | 0 | 1 | 2 | 3 | 4 |
| 11 | Me insulto a mí mismo/a. | 0 | 1 | 2 | 3 | 4 |
| 12 | Creo que merezco mis pensamientos críticos hacia mí mismo. | 0 | 1 | 2 | 3 | 4 |
| 13 | Hay una parte de mí mismo/a que quiere deshacerse de las partes de mí que no me gustan. | 0 | 1 | 2 | 3 | 4 |
| 14 | Me animo de cara al futuro. | 0 | 1 | 2 | 3 | 4 |
